# Tennis injury data from The Championships, Wimbledon, from 2003 to 2012

**DOI:** 10.1136/bjsports-2015-095552

**Published:** 2016-01-11

**Authors:** I McCurdie, S Smith, P H Bell, M E Batt

**Affiliations:** 1 Defence Medical Rehabilitation Centre (DMRC) Headley Court, Epsom, Surrey, UK; 2 All England Lawn Tennis Club, London, UK; 3 Queens Medical Centre, Nottingham, UK; 4 The Paddocks Clinic, Princes Risborough, UK; 5 Nottingham University Hospitals NHS Trust, Nottingham, UK; 6 Arthritis Research UK Centre Sport, Exercise and Osteoarthritis, Nottingham, UK

**Keywords:** Injury, Cohort study, Retrospective, Tennis

## Abstract

**Objective:**

The primary aims of this retrospective study were to describe the burden of injury presenting to the medical team and the changes in injury profile over 10 years (2003–2012) at The Championships, Wimbledon. Secondary aims included description of gender difference in rates, distribution and pathology of injuries.

**Design:**

Retrospective observational cohort of player injury presentations over 10 years (2003–2012) at The Championships, Wimbledon.

**Results:**

The overall rate of presentation of injury for all players over the 10-year period was 20.7 per 1000 sets played. Injury rates were lower for male players (17.7 injuries per 1000 sets played) than female players (23.4 injuries per 1000 sets played). There was variability in the numbers of injuries reported by men and women players over the 10-year period.

**Conclusions:**

The rates of presentation of injury at this Grand Slam tennis tournament varied between male and female players, and between years. More robust systems of data collection are required in professional tennis to enable more sophisticated injury data analysis between sexes, years and different playing surfaces.

## Introduction

There is a paucity of published data describing the incidence of injury in elite tennis. Within the published data there is considerable variability in the reported incidence of injuries, ranging from 0.04 to 3.0 injuries per 1000 playing hours in players of all ages.^1^ It is widely acknowledged that further studies are required, particularly involving professional tennis players.^2^

Previous tennis epidemiological reviews have found that acute injuries more typically occur in the lower limbs, whereas chronic overuse injuries occur more frequently in the upper limbs and trunk.^2^ Musculoskeletal injuries in tennis can affect almost any part of the body, with the majority of injuries being classified as overuse injuries resulting from repetitive microtrauma.^3^ Gender is not thought to influence injury rate.^2^ Identification of the site at risk of injury and associated factors contributing to the risk of injury can help target more specific injury prevention strategies to maximise player health and minimise injury risk.

The primary aims of this retrospective study were to report the rates of presentation of injury and the changes in injury profile over 10 years (2003–2012) at The Championships, Wimbledon. Secondary aims included description of gender difference in rates of presentation, distribution and pathology of injuries.

## Methods

The Championships, Wimbledon, started in 1877 and is the oldest of the four Grand Slam tennis tournaments and the last to be played solely on grass courts. It is held at the All England Lawn Tennis Club (AELTC) over 3 weeks in June and July each year. The main draw lasts 14 days and is made up of men's singles, ladies’ singles, men's doubles, ladies’ doubles and mixed doubles.

This retrospective cohort study includes injury presentations recorded at The Championships, Wimbledon, over a 10-year period from 2003 to 2012. Medical care for the players at The Championships is provided by a multidisciplinary team that includes a general practitioner who manages the majority of non-injury conditions, alongside a team of sports physicians who deal with all musculoskeletal injuries. Data were collected on all presentations of injury to the sports physicians throughout the main draw period (follow-up consultations were not included). These presentations include court calls, referrals from the tournament physiotherapists (AELTC, Association of Tennis Professionals (ATP) and Women's Tennis Association (WTA)) and less frequently from self-referral. For the purposes of this study, injuries to junior and senior players were excluded, as were data from non-players such as coaches and support staff. Data were anonymised and injuries coded with Orchard Codes V.8. For the period from 2007 to 2012, data were also available about the type of injury (overuse or trauma) and the time scale of the injury, that is, acute new, acute prior, chronic or recurrent as defined below.

### Exposure

In the consensus statement on epidemiological studies of medical conditions in tennis it was argued that exposure should be calculated using the duration of play.^4^ Detailed exposure data may be collected within prospective study designs, albeit typically missing practising hours. For retrospective data, a surrogate for exposure may be used, such as match exposures or number of sets played.^5^ For this study we calculated the number of sets played per year for singles, doubles and mixed doubles (singles counted as 2 player exposures and doubles counted as 4 player exposures per set) and expressed the rate of injury as the number of injuries per 1000 sets played. The total number of sets played through the Championships main draw is reasonably consistent across the study period with a mean of 1221 (range 1141–1258). This represents a total of 33 790 ‘player sets’ (see table 1) with an annual mean of 3379 (range 3070–3546). Each year, the men play an average of 2011 sets and the ladies 1368 sets.

**Table 1:** Numbers of sets played at The Championships, Wimbledon, 2003–2012

Year	Number of sets played at The Championships	Number of player sets*		
		Men	Ladies	Total
2003	1245	2040	1450	3490
2004	1141	1736	1334	3070
2005	1221	2006	1378	3384
2006	1224	2054	1344	3398
2007	1223	2048	1360	3408
2008	1218	2002	1358	3360
2009	1225	2046	1336	3382
2010	1258	2136	1364	3500
2011	1187	1872	1380	3252
2012	1270	2170	1376	3546
Total	12 212	20 110	13 680	33 790
Mean	1221	2011	1368	3379

*Calculated as two player sets per singles match and four player sets per doubles match.

New presentations to the sports physicians were defined as being acute, recurrent or chronic. Acute injuries were further classified as being ‘acute new’ (occurring during The Championships) or ‘acute prior’ (occurring at some point before arriving at Wimbledon, eg, during a previous tournament). Those injuries occurring within 8 weeks of recovery from a similar injury to same site were classified as recurrent. Those injuries that were pre-existing and ongoing prior to the start of the tournament were classified as chronic.

### Analysis

Owing to the retrospective nature of these data and injuries included that occurred prior to The Championships, formal analysis of injury incidence was considered inappropriate. The numerator data include all musculoskeletal presentations to the sports physicians during the 2-week period of The Championships Main Draw. The denominator is the number of sets of tennis played during this same period. As the numerator includes players presenting with injuries occurring both before and during The Championships, it should be stressed that these data do not represent the true incidence of injury occurrence during play through The Championships. They do, however, provide the reader with a realistic and practical insight into the burden of and trends in musculoskeletal presentations at a Grand Slam tennis tournament over a 10-year period.

## Results

**Figure 1:**
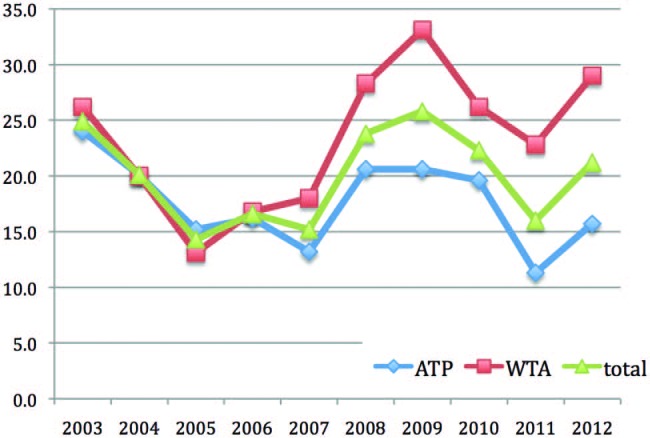
Rates of injury per 1000 sets played at The Championships, Wimbledon, 2003–2012. ATP, Association of Tennis Professionals; WTA, Women's Tennis Association.

A total of 700 injuries were seen over 10 years during the main draw at The Championships, Wimbledon, with a total of 12 212 sets played. There was an average of 3379 sets of player exposure each year over this 10-year period (see table 1), equating to an overall rate of 20.7 (range 14.3–25.8) injuries per 1000 sets played (figure 1).

**Figure 2:**
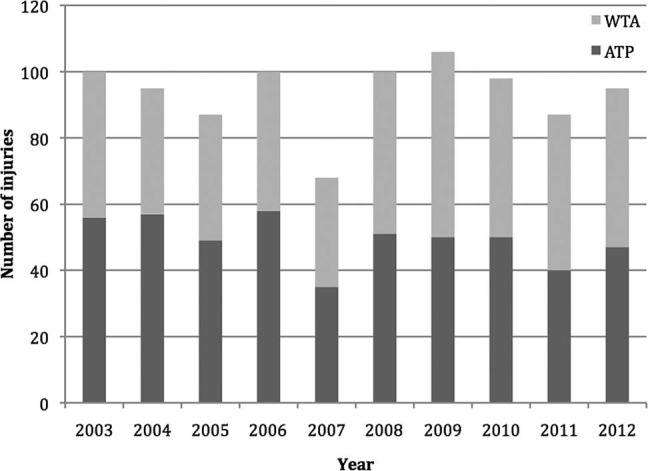
The male/female distribution of injuries over the 10 years. ATP, Association of Tennis Professionals; WTA, Women's Tennis Association.

Injury rate was lower for male players (17.7 injuries per 1000 sets played) than female players (23.4 injuries per 1000 sets played). There was variability in the numbers of injuries reported by men and women players over the 10-year period (figure 2).

**Figure 3:**
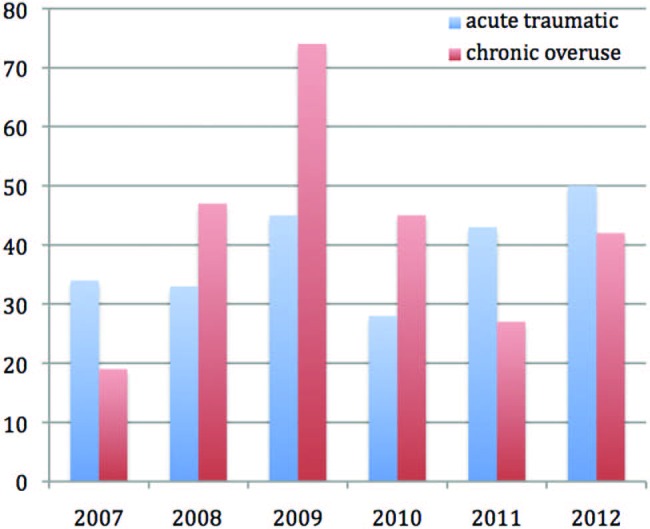
Injury classification by category: 2007–2012.

Over a 6-year period (2007–2012), presentations were further categorised into trauma and overuse in an attempt to better understand aetiology. Over this period, 233 (48%) presentations were from a traumatic episode and 254 (52%) had arisen from overuse (figure 3).

**Figure 4:**
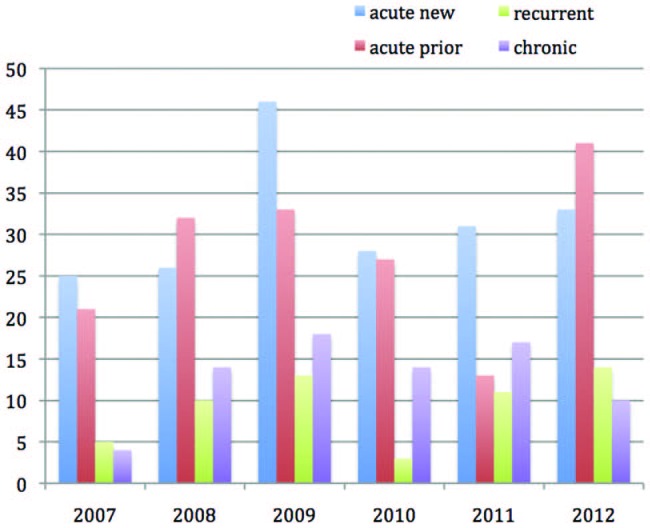
Injury classification by type of injury: 2007–2012.

A further subclassification of the timing of onset of injury was used over these 6 years (figure 4). Thirty-nine per cent of injuries were acute new presentations occurring during The Championships, while 34% presentations related to acute traumatic injuries that had been sustained elsewhere prior to The Championships (acute-prior). Sixteen per cent of presentations were pre-existing chronic in nature and a further 11% injuries had occurred within 8 weeks of recovery from a similar injury to same site and were classified as recurrent injury.

**Figure 5:**
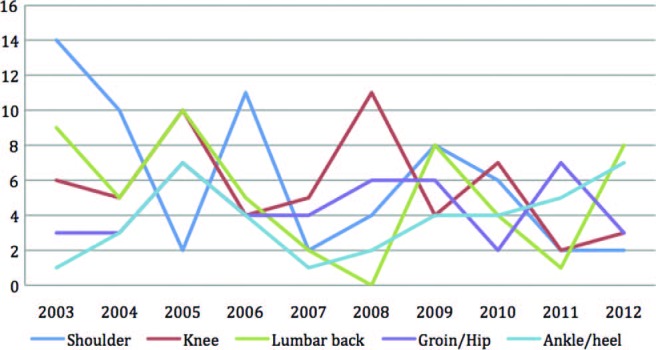
Five most common anatomical sites of pathology in male tennis players.

**Figure 6:**
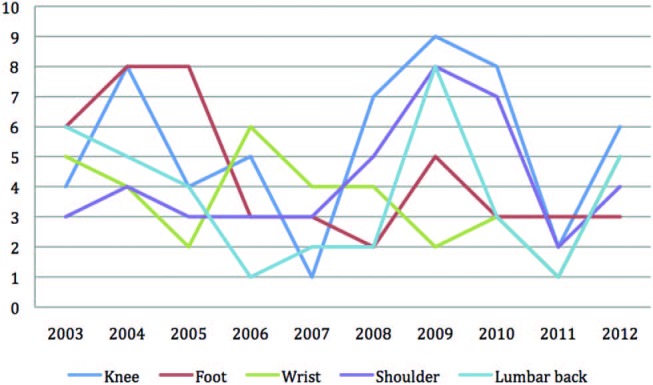
Five most common anatomical sites of pathology in female tennis players.

**Figure 7:**
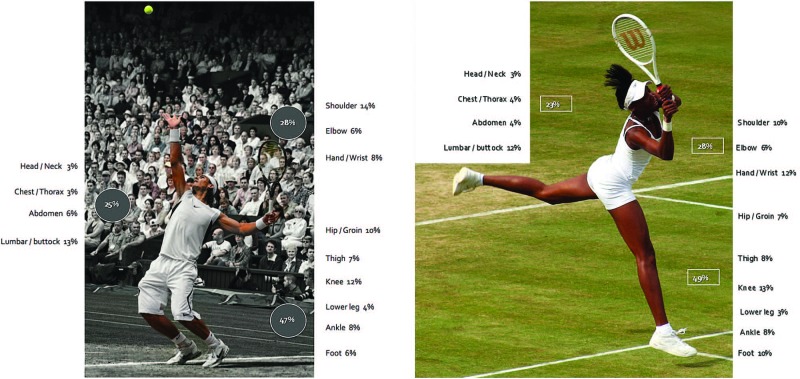
Distribution of injuries in male and female players.

Figures 5 and 6 display the commonest anatomical sites of injury for male and female tennis players over the 10-year period. There is variability between years and between male and female players, but the small numbers of injuries at each anatomical site over a year does not permit analysis of injury trends. Nonetheless, shoulder, knee and lumbar spine presentations are common in both male and female tennis players at The Championships. Male players appear to sustain more groin, hip, ankle and heel injuries, with wrist and foot problems being commoner in female players. Figure 7 shows a graphic representation of the proportion of injuries sustained by male and female players by body region over the 10-year period, 2003–2012. Axial injuries (including head, spine and abdomen) account for 25% and 23% injuries in male and female players, respectively, while upper limb injuries account for 28% injuries in both genders and lower limb injuries account for 47% and 49% injuries in male and female players, respectively.

**Figure 8:**
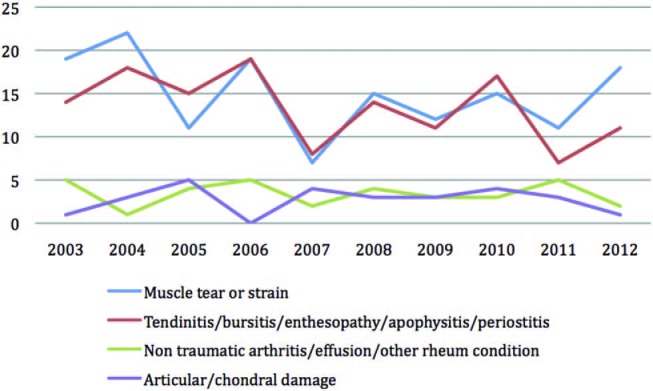
Type of pathology of injury in male players.

**Figure 9:**
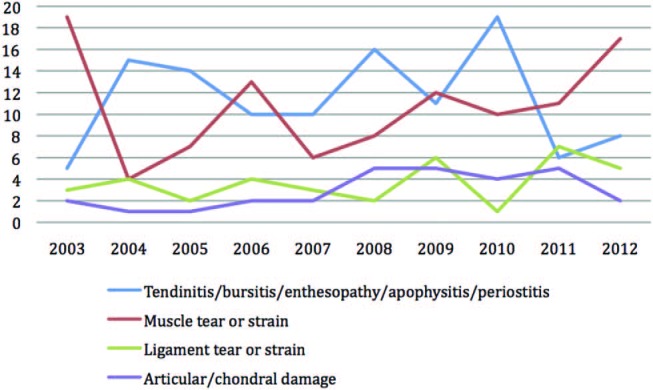
Type of pathology of injury in female players.

Using Orchard codes we were able to classify injuries by tissue pathology for male and female players. Male players have equal rates of muscle strain/tear as they do tendinopathy, whereas female players seem to suffer relatively fewer muscle than tendon injuries (figures 8 and 9).

## Discussion

There is a dearth of data in the literature describing the incidence and pattern of injury in professional tennis, as well as a lack of consistency in the collection and reporting of these data. As a result, little is known about the morbidity relating to injury among professional players on the ATP and WTA tours and when playing Grand Slams.

Efforts to report injury in competitive tennis have largely been confined to cross-sectional studies of specific subgroups, with a focus on juniors.^6–8^ Injuries may be self-reported and in some cases lack exposure data, resulting in an absence of injury incidence data. Longitudinal data have recently been reported for illness and injury among players at the US Open.^5^ ^9^ Our data add to this body of knowledge by describing the injury profile among elite tennis players playing on grass over a 10-year period, covering a total exposure of 33 790 player sets of tennis. The data are reported in terms of injuries per 1000 sets of match play tennis. While there is no consistent reporting method, a recent Consensus Statement advocates using ‘per 1000 h’ as the preferred denominator.^4^ The nature of retrospective data analysis makes this difficult, since a record of the actual numbers of hours in match play are required for each player. The alternative use of ‘per 1000 sets’ may seem less scientifically robust, but is more workable for reporting injury rates in professional tennis.

A secondary aim of this study was to better understand the volume and case mix of injuries seen at a major Grand Slam tournament. Tennis injuries appear to affect a wide range of anatomical sites that reflect the nature of a sport in which most elements of the kinetic chain are stressed to the limit. Most studies have reported no differences in incidence between male and female players,^1^ although it is interesting to note that although male players have a slightly lower injury rate they play 50% more sets than the female players (table 1). The variation in distribution of injury between male and female players reported here may reflect differences in both style of play and match duration between the male and female grass game (men's matches last on average nearly 1 h longer). Further studies are needed to establish if there are any meaningful differences in injury pattern between the sexes.

The distribution and type of injuries seen in our data are very similar to those reported by Sell *et al*^9^ with 48% injuries affecting the lower limbs and muscle injury being slightly more common in male players. Comparison with other tennis tournaments is, however, limited by a number of significant confounding variables, such as time of year (in relation to environment and tennis season) and court surface. Also of interest is the potential effect of court surface on injury incidence and profile. While our data are not able to support or refute the notion that playing on grass courts (or perhaps the switch from clay to grass) is a significant risk factor for injury, it might seem reasonable to suspect that play on the faster grass court surface with a lower ball bounce and shorter point duration might influence injury patterns. The current professional tennis season demands that players switch between all three court surfaces within very short time frames and challenges their ability to adapt and continue to perform without sustaining injury. Andre Agassi, in his autobiography ‘Open’^10^ writes “the sudden switch from one surface to another changes everything. Clay is a different game, thus your game must become different, and so must your body.” The finding of a significant proportion of injuries that were sustained prior to arrival at The Championships (61%: including acute prior, chronic and recurrent injuries) is an indication of the punishing nature of the professional tennis season.

Perhaps the greatest challenge to our understanding of injury profiles in elite tennis is the lack of consistent injury and exposure data. Recent initiatives by the International Tennis Federation (ITF) and the ATP/WTA Tours have seen the implementation of a centralised electronic record keeping system, which should enable consistent data collection and reporting using standardised definitions and methodology.^11^ Data from all healthcare professionals involved with the management of players on the world tour are entered onto the system and should ultimately become available for more detailed analysis. Such data should enhance our understanding of the true extent and breadth of injury in professional tennis and the significance of various potential risk factors for injury, such as the court surface and changing between surfaces during the competitive season. Furthermore, these data should enable more effective continuity of care of players throughout the competitive season. Opportunities to improve the knowledge and skills of clinicians working with tennis players and to develop effective, evidence-based injury prevention strategies may then become a realistic possibility.

### Study limitations

The data reported here are retrospective and represent only those injuries that were brought to the attention of the Sports Medicine team at The Championships. Given that the ATP/WTA/AELTC and individual players’ personal therapists do manage some conditions without Sports Physician input, the total burden of injury will be higher than reported here. The threshold for seeking Sports Medicine input may vary between players and the different groups of physiotherapists according to organisational policy and/or personal practice and this could introduce an element of referral bias.

**What are the findings?**

- These data are the first to describe the injuries presenting among professional tennis players during the 2 weeks of a major grass court tennis event over a 10-year period.
- The majority of injuries presenting at this tennis Grand Slam competition were pre-existing or recurrent, that is, occurred prior to arrival at Wimbledon.
- Muscle and ligament injuries are the predominant type of acute injury in professional grass court tennis and, despite annual variability, appear to have increased in female players during the study period.
- Ligament and articular surface injuries are less common and vary little from year to year.

**How might it impact on clinical practice in the future?**

- Decisions on safe return to play following injury may be informed by these data, which illustrate the high prevalence of unresolved or recurrent injuries among professional tennis players in competition.
- Standardisation and consistency of injury and exposure data across the professional tennis circuit are critical to the understanding of injury management and the continuity of clinical care.

## References

[1] Pluim BM, Staal JB, Windler GE, et al Tennis injuries: occurrence, aetiology, and prevention. Br J Sports Med 2006;40:415–23. 10.1136/bjsm.2005.023184 16632572 PMC2577485

[2] Abrams GD, Renstrom PA, Safran MR Epidemiology of musculoskeletal injury in the tennis player. Br J Sports Med 2012;46:492–8. 10.1136/bjsports-2012-091164 22554841

[3] Ellenbecker TS, Pluim B, Vivier S, et al Common injuries in tennis players: exercises to address muscular imbalances and reduce injury risk. Strength Cond J 2009;31:50–8. 10.1519/SSC.0b013e3181af71cb

[4] Pluim BM, Fuller CW, Batt ME, et al Consensus statement on epidemiological studies of medical conditions in tennis, April 2009. Br J Sports Med 2009;43:893–7. 10.1136/bjsm.2009.064915 19900956

[5] Sell K, Hainline B, Yorio M, et al Illness data form the US Open Tennis Championships from 1994 to 2009. Clin J Sports Med 2013;23:25–32. 10.1097/JSM.0b013e31826b7e52 23011554

[6] Winge S, Jorgenson U, Nielson L Epidemiology of injuries in Danish championships tennis. Int J Sports Med 1989;10:368–71. 10.1055/s-2007-1024930 2599726

[7] Kovacs MS, Ellenbecker TS, Kibler WB, et al Injury trends in American competitive junior tennis players. J Med Sci Tennis 2014;19:19–23.

[8] Silva RT, Takahashi R, Berra B, et al Medical assistance at the Brazilian juniors tennis circuit. J Sci Med Sport 2003;6:14–18. 10.1016/S1440-2440(03)80004-X 12801206

[9] Sell K, Hainline B, Yorio M, et al Injury trend analysis from the US Open Tennis Championships between 1994 and 2009. Br J Sports Med 2014;48:546–51. 10.1136/bjsports-2012-091175 22923462

[10] Agassi A. *Open*. Plon, 6 Jan 2011. Jan 6.

[11] Pluim BM The evolution impact of science in tennis: eight advances for performance and health. Br J Sports Med 2014;48:i3–5. 10.1136/bjsports-2014-093434 24668376 PMC3995242

